# Model-based analysis of two-color arrays (MA2C)

**DOI:** 10.1186/gb-2007-8-8-r178

**Published:** 2007-08-29

**Authors:** Jun S Song, W Evan Johnson, Xiaopeng Zhu, Xinmin Zhang, Wei Li, Arjun K Manrai, Jun S Liu, Runsheng Chen, X Shirley Liu

**Affiliations:** 1 Department of Biostatistics and Computational Biology, Dana-Farber Cancer Institute, 44 Binney Street, Boston, MA 02115, USA; 2 Department of Biostatistics, Harvard School of Public Health, Boston, MA 02115, USA; 3 Bioinformatics Laboratory, Institute of Biophysics, Chinese Academy of Sciences, Beijing 100101, China; 4 NimbleGen Systems, Inc., Science Court, Madison, Wisconsin 53711, USA; 5 Department of Physics, Harvard University, Cambridge, MA 02138, USA; 6 Department of Statistics, Harvard University, 1 Oxford Street, Cambridge, MA 02138, USA

## Abstract

A novel normalization method based on the GC content of probes is developed for two-color tiling arrays. The proposed method, together with robust estimates of the model parameters, is shown to perform superbly on published data sets. A robust algorithm for detecting peak regions is also formulated and shown to perform well compared to other approaches. The tools have been implemented as a stand-alone Java program called MA2C, which can display various plots of statistical analysis for quality control.

## Abstract 2

A normalization method based on probe GC content for two-color tiling arrays and an algorithm for detecting peak regions are presented. They are available in a stand-alone Java program.

## Background

**Figure 1:**
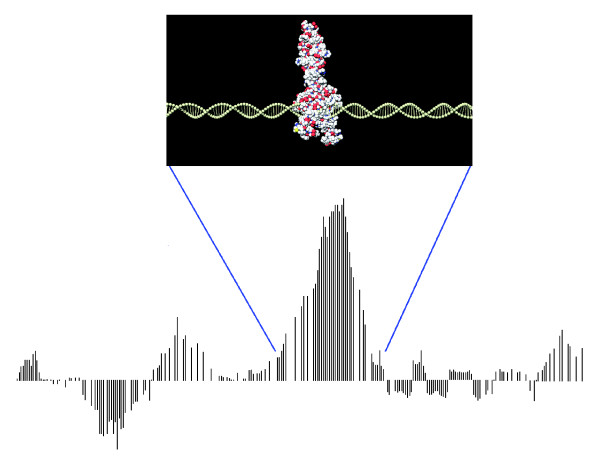
ChIP-chip. Regions of interest on DNA are densely tiled, with probes separated by short distances. In this figure, each bar corresponds to the log-ratio hybridization signals of two channels measured by a probe. Small sub-regions that are over-represented compared to the genomic background will appear as pronounced peaks (in this example, the middle peak represents the DNA fragments containing a protein-binding site). The computational challenge is to normalize the data properly and to detect confident enriched regions by filtering out false peaks (left and right peaks in this example).

High-density oligonucleotide tiling-microarrays currently provide the most powerful method of investigating genome-wide protein-DNA interactions and chromatin structure *in vivo*. As illustrated in Figure 1, the technology allows tiling regions of interest on DNA with probes separated by short chromosome distances. A typical NimbleGen array has about 400,000 probes that are 40-60 nucleotides long and separated by 10-100 base-pairs (bp) in the genome. Both NimbleGen and Agilent provide two-color microarrays with flexible designs where one can choose probes that are partially overlapping for high resolution studies of chromatin structure. The experimental protocol requires labeling the treatment and control samples with fluorescent dyes, usually green and red, and then hybridizing them on a microarray. Each probe's intensity of fluorescence upon scanning the microarray will give an approximate measure of the abundance of DNA that hybridized to the probe. Because each probe has an associated genomic coordinate, one can plot the intensities as a function of chromosome locations and then reconstruct the enrichment of particular DNA or RNA fragments compared to the genomic background. As in Figure 1, the enriched regions appear as peaks, which can represent protein-bound DNA fragments.

The technology is continuing to develop rapidly, but certainly not without difficulties that are imposed by the inherent complexity of biological systems and, as such, must be addressed by computational means for the foreseeable future. The main computational challenge lies in properly normalizing the data and distinguishing true peaks from the noisy background. Many problems that confound this type of microarray data actually arise from probe-specific biases, such as differential sequence copy numbers in the genome or variable melting temperature dependent upon the GC content. For Affymetrix tiling arrays, several good model-based methods already exist to account for probe biases and, thus, to adjust for probe-specific baseline signals. The recently introduced MAT [1], for instance, estimates probe affinity from probe sequence and copy number and provides a powerful tool for finding enriched regions in chromatin immunoprecipitation (ChIP) and other applications on Affymetrix tiling-array experiments. Incidentally, similar problems are also found in Affymetrix expression arrays, for which extensive effort has been previously exerted by various groups to develop robust methods for background correction and probe-level normalization (for example, [2-5]). It is relatively hard and expensive for Affymetrix to provide custom designed microarrays.

Commercial custom tiling arrays are relatively new in the field of microarray biotechnology and, just as expression arrays allow global assays of gene expression, provide an invaluable tool for investigating the locations and roles of DNA-binding proteins in the whole genome at high resolution. All currently available custom tiling arrays use the two-color technology. Considering the utility and power of high-resolution tiling arrays, it is thus imperative that reliable computational methods be developed now to facilitate the extraction of precise and accurate conclusions from such experiments.

**Figure 2:**
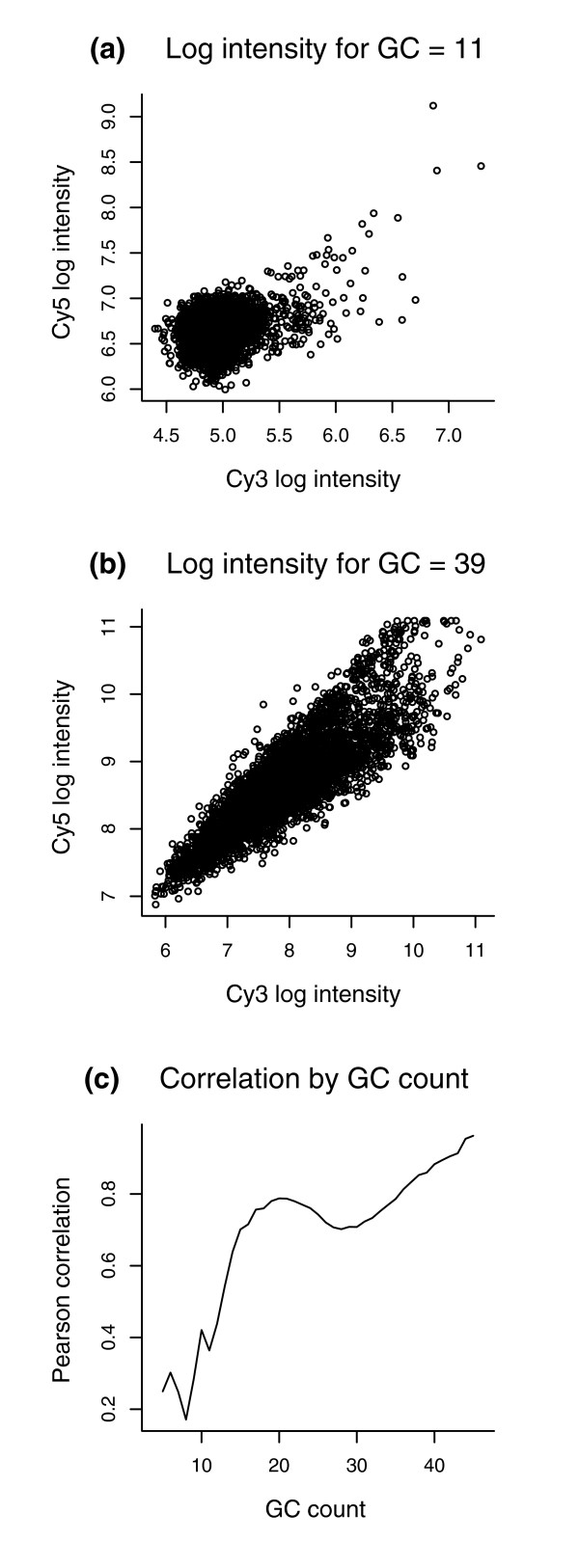
Scatter plots of the Cy5 versus Cy3 channels for 50-mer probes from [12] with **(a) **28256_Input versus 28256_ChIP for *G*+*C *= 11 bases and **(b) **28256_Input versus 28256_ChIP for *G*+*C *= 39 bases. The correlation is 0.364 in (a) and 0.860 in (b). **(c) **Plot of the inter-channel correlation (28256_Input, 28256_ChIP) across GC bins within the same array. The higher GC-count probes are more correlated and, therefore, should be more reliable in detecting differentially expressed or enriched probes. That is, in ChIP-chip, more than 99% of probes just measure the background and, thus, should ideally give similar results for the two channels. The correlation between the two channels, however, depends on the GC content of the probes. Since the two-channel correlation for high-GC probes is much higher than that for low-GC probes, significant two-channel fold-changes in the former category are much more reliable than those in the latter category, where large fold-changes may readily occur by chance.

**Figure 3:**
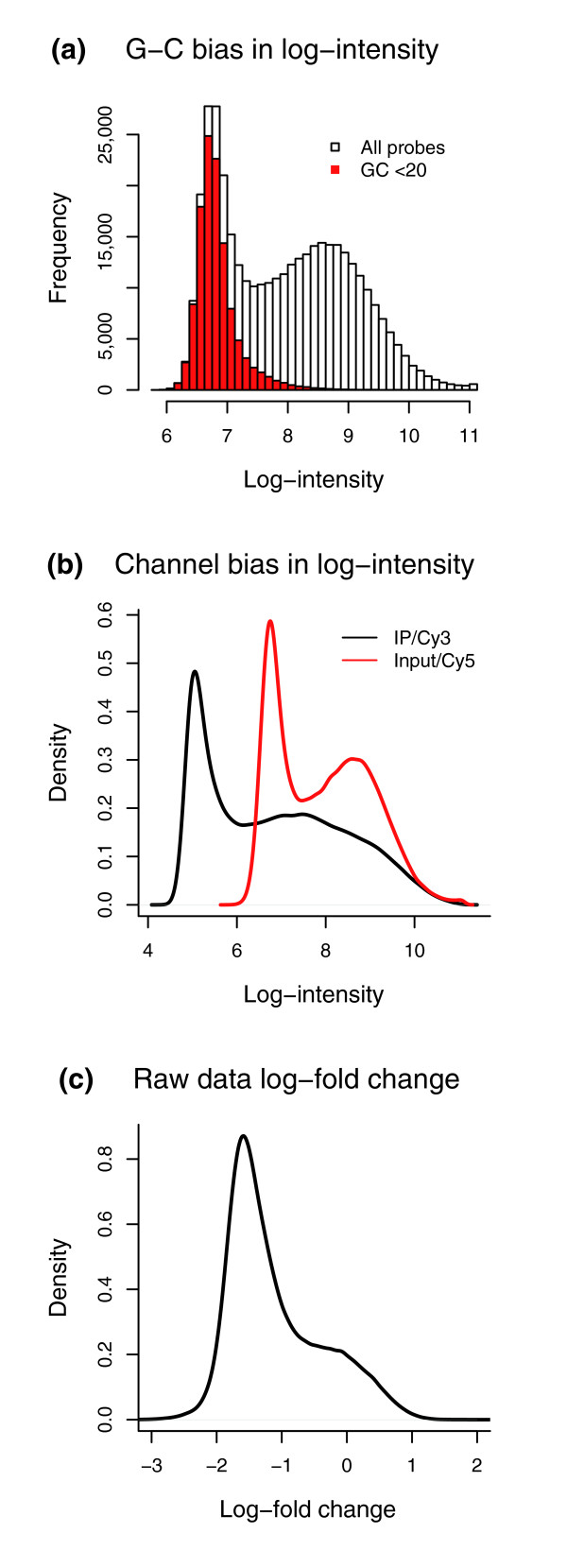
Histograms of intensities. **(a) **Histogram of single-channel log-intensity values for a single array from 28256_Input [12]. The red bars represent the log-intensities for the probes with *G*+*C *less than 20, indicating that the bimodal behavior is caused by the GC content of probes. **(b) **Density plot of single channel log-intensities for two channels on the same array (28256_ChIP, black; 28256_Input, red). Notice that both the scale and the mean of the individual channels must be adjusted to properly normalize the arrays. **(c) **The raw data log-ratio values (28256_ChIP/28256_Input) for the same array in (b). Note that the 'bump' at 0 is not caused by enrichment but by lack of channel specific normalization of the data.

It turns out that two-color arrays also exhibit a sequence bias, particularly dependent upon the GC content of probes. More precisely, probes with high GC counts tend to have high intensity; furthermore, as Figure 2 indicates, the two channels show a higher correlation in the high-GC probes than in the low-GC probes. However, no satisfactory normalization and peak-detection methods are yet available for two-color tiling arrays. For example, even though NimbleGen provides flexible custom designs, with long probes to minimize cross-hybridization and variable probe spacing to allow dense tiling, a robust method of analysis has not been hitherto developed for the platform. Indeed, NimbleGen currently uses a simple method of globally scaling all probe ratios by the median, attempting to remove any dye-bias across arrays but neglecting other probe-specific biases. As illustrated in Figure 3, the median scaled ratios retain the bimodal distribution attributable to GC probe effects and, thus, this approach is inadequate in removing all dye and sample biases from the data.

For dual-channel cDNA arrays, several normalization methods have been proposed (for example, [2,6]), but these procedures typically utilize methods that neglect probe sequence information and are also computationally expensive and, thus, unsuitable for currently available high-density tiling arrays. One common way of locally normalizing two-color arrays is the so-called *M*-*A *loess normalization. The fundamental assumption behind this procedure is that most probes should have similar values between the two-channels, an assumption violated in studies of chromatin structure such as nucleosome mapping described in [7,8]. This method also does not account for sequence-specific effects, which may be significant in high-density tiling arrays, and also does not normalize the variance of *M*.

Single-channel normalization methods can be also applied to two-color arrays, such as those proposed by [3,9], but they ignore the fact that the two channels are paired, and such approaches are thus likely to retain residual effects or correlation. Recently, Dabney and Storey [10] have introduced a normalization method that adjusts for intensity-dependent dye bias and array-to-array variations. However, their method, which was developed for expression arrays, does not model sequence-specific probe effects and is based on smoothing procedures that can be computationally demanding for tiling arrays; the approach also requires a dye swap and, thus, cannot be applied to single array experiments, which are often performed as test runs. In fact, as far as we are aware, there are, to date, only two published tools, MPeak [11,12] and ChIPOTle [13,14] for analyzing two-color high-density tiling arrays, but neither considers probe-specific normalization or is able to combine replicate experiments directly. This problem is rather serious since biological replicate experiments are perceived to be indispensable in any sound research utilizing microarrays.

In this paper, we address many of the issues discussed above and present robust algorithms for normalizing the raw data at probe-level and detecting peaks, implemented as a Java program called MA2C (model-based analysis of two-color arrays). Because our normalization method standardizes the probe intensities, our peak-detection algorithm naturally generalizes to combine replicate arrays.

## Results and discussion

### Comparison of normalization methods

**Figure 4:**
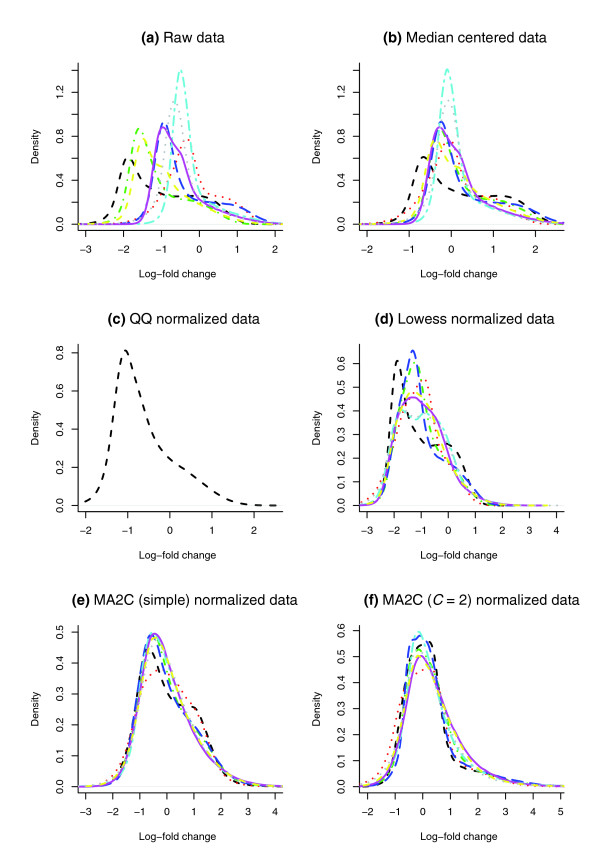
Log-ratio density plots. All samples are from [12]: **(a) **raw data; **(b) **median adjusted data; **(c) **QQ normalized data; **(d) **Lowess normalized data; **(e) **MA2C (Simple) normalized data; **(f) **MA2C (Robust *C *= 2) normalized data. Different colors correspond to different samples.

To test the effectiveness of the MA2C normalization procedure, we compared the MA2C normalized data using the non-robust and robust *C *= 2 methods with the raw and median scaled log-ratio data; Figure 4 shows the corresponding density plots of log ratios for eight samples published in [12]. Figure 4 illustrates that our method standardizes the data much more effectively than median scaling and removes much of the GC-effect discussed in Figure 3. In particular, Figure 4d shows that the log-ratios normalized with MA2C's robust option follow a normal distribution.

### Spike-in experiment

We used the data (GEO GSE7523) from a recent spike-in experiment to test MA2C. The spike-in samples contained 96 clones in the ENCODE region of approximately 500 bp, at 8 different concentrations corresponding to (2^n ^+ 1)-fold enrichment compared to the human genomic DNA, for *n *= 1,...,8, and 12 different clones per concentration. The control sample contained sonicated genomic DNA without spike-ins. The spike-in and control samples were differentially labeled and hybridized to a NimbleGen ENCODE tiling array in triplicates, and the resulting data were used to assess the performance of MA2C against other currently available algorithms.

MA2C and MPeak Version 2.0 [11,12] were run using default parameters, and ChIPOTle v1.0 [13,14] using window size 500, step length 100, *p *value cutoff 10^-4 ^and Gaussian background distribution. As seen in Table 1, while having a comparable sensitivity, MA2C has a higher positive predictive value and, thus, fewer false negative peaks than ChIPOTle. After removing ambiguous overlapping regions from the 96 spike-in regions, we used the remaining 47 unique regions to measure the correlation between spike-in fold-changes and the corresponding algorithm-assigned scores for detected peaks. MA2C not only found all the unique sites but also showed a better correlation than ChIPOTle, which missed some of the sites in the first sample.

**Table 1:** Comparison of MA2C with other algorithms using a spike-in experiment with a total of 96 regions and 47 unique non-overlapping regions

Algorithm	CHIP_ID	PPV	Sensitivity	Unique	Correlation
ChIPOTle	49875	71%	85%	40	0.72
	49880	69%	98%	47	0.76
	49883	73%	98%	47	0.79
MPeak	49875	100%	91%	46	0.74
	49880	96%	89%	46	0.71
	49883	98%	89%	46	0.79
MA2C	49875	99%	91%	47	0.78
(*C *= 2 normalized)	49880	96%	94%	47	0.79
	49883	99%	95%	47	0.81
	All 3	96%	96%	47	0.81
MA2C	49875	99%	92%	46	0.77
(Global median-scaled)	49880	100%	93%	46	0.79
	49883	99%	92%	46	0.81
	All 3	100%	95%	47	0.80

PPV (positive predictive value) = no. of true positive peaks/no. of total peaks. Sensitivity = no. of detected true positive regions/96. Unique = number of unique regions found. Correlation = correlation coefficient of the spike-in log fold-changes and algorithm-assigned scores for the 47 unique regions.

The positive predictive value of MPeak was comparable to MA2C, but MA2C was more sensitive and also found more unique sites. MA2C again showed a better correlation with spike-in fold-changes than MPeak and, thus, provided better quantitative information about the enriched regions than both ChIPOTle and MPeak. We also tested the MA2C peak detection algorithm on the global median-scaled data without any GC-correction (the same data analyzed with MPeak and ChIPOTle) and still found MA2C to be more sensitive and to have a higher positive predictive value, indicating that MA2C can outperform other available algorithms even without its GC-specific normalization step (Table 1).

Furthermore, neither MPeak nor ChIPOTle can combine replicate data in a single test. As seen in Table 1, pooling data from replicate experiments can often increase the sensitivity and quantitativeness of analysis, and this option implemented in MA2C will prove to be useful. Since ChIP-chip experiments require biological replicates, which are much noisier than the technical triplicate spike-ins presented here, the ability to combine replicates at the probe-level will provide more sensitive and robust peak predictions than other methods of combining peaks. In addition, ChIP-chip experiments contain a PCR amplification step that often increases the GC bias of probes; in this regard, MA2C's GC-based probe normalization shows distinct advantages over ChIPOTle and MPeak on PCR amplified samples, as observed in a separate PCR amplified spike-in experiment (unpublished data).

### ChIP-chip data in *Caenorhabditis elegans*

The protein DPY-27 functions as an essential dosage-compensator that suppresses the expression of genes on each X chromosome in hermaphrodite XX embryos of *Caenorhabditis elegans*, thereby reducing the expression level of the X-linked genes by half to the level in XO (male) counterparts. Chuang *et al*. [15] have shown that the basic suppression mechanism involves localization of DPY-27 to X chromosomes, likely leading to a subsequent modification of the chromatin structure of X chromosomes mediated by DPY-27. Davis and Meyer [16] later showed that SDC-3 also localizes to X chromosomes in XX hermaphrodites and associates with a dosage compensating complex involving DPY-27.

A recent study [17] suggests that SDC-3 in fact preferentially binds in the promoter regions of active genes. This observation has the important biological implication that SDC-3 and DPY-27 may modulate transcriptional activities and that the mechanism by which the dosage compensating complex spreads along the X chromosome may involve initial localization to promoters followed by RNA polymerase-coupled dispersion. Their conclusion thus relies on the fact that a significant fraction of the total SDC-3 binding sites resides in proximal promoter regions. We tested MA2C and MPeak on their triplicate data to see whether we can improve the fraction and number of SDC-3 binding sites in promoters - a finding that could strengthen the claim made in [17]. We compared the results with the ChIPOTle analysis provided to us by Ercan *et al*. [17]; as previously mentioned, ChIPOTle cannot directly combine replicate experiments, so the authors first found peaks from median *z*-scores and selected the peaks that occur in two of the three replicates. It should be noted that the number of SDC-3 binding sites quoted here is different from that reported in [17] because, in that paper, the peaks that appeared in negative control experiments without antibody were removed from the list. We ran MA2C using a window-size of 600 bp at *p *value cutoffs of 10^-5 ^and 10^-4^; all other parameters were set to default settings. MPeak was run using default parameters. As seen in Table 2, compared to both programs, MA2C could find not only a greater number but a higher fraction of SDC-3 binding sites in promoter regions, further strengthening the conclusion propounded in [17]. In addition, Table 3 shows that MA2C can also detect almost all the regions found by ChIPOTLe and MPeak. MA2C's high sensitivity and power can thus provide a valuable tool for discovering novel biological phenomena.

**Table 2:** Numbers and annotation of SDC-3 binding sites detected by different methods

Algorithm	Sample	No. of peaks	In promoter
ChIPOTle	Combined triplicate	1,219	33.63%
MPeak	Replicate 1	1,819	30.35%
	Replicate 2	921	29.32%
	Replicate 3	557	34.11%
MA2C	Combined triplicate (*p *= 10^-5^)	1,181	38.5%
MA2C	Combined triplicate (*p *= 10^-4^)	1,588	35.1%

For annotation, promoter regions 1 kb upstream from translation start sites of genes were used, because the annotation of transcription start sites in *C. elegans *has not yet been well established.

**Table 3:** Overlap of binding sites of SDC-3

	ChIPOTle	MPeak 1	MPeak 2	MPeak 3	MA2C (*p *= 10^-5^)
ChIPOTle	100%	65.97%	87.08%	92.28%	67.06%
MPeak 1	56.69%	100%	17.26%	26.57%	68.25%
MPeak 2	37.16%	8.74%	100%	21.01%	36.07%
MPeak 3	25.84%	8.14%	12.70%	100%	24.72%
MA2C (*p *= 10^-5^)	97.54%	97.91%	98.05%	99.64%	100%

Percentages of SDC-3 binding sites from a method in columns overlapping with those from a method in rows (MPeak 1 denotes MPeak results from replicate 1, and so forth; two regions were considered to be overlapping if they shared at least 1 bp).

## Conclusion

### Novel applications

ChIP-chip technology has quickly become popular among biologists, and high-density tiling microarrays are increasingly being used in novel genomic research. Some of the interesting applications involve finding novel transcripts in the genome, DNA methylation sites, nucleosome positions, DNA hypersensitivity regions, and alternative splicing events [7,8,18-21].

In all of these studies, which tend to combine experiments performed at various time points and under different conditions, the variability of array performance and sequence-specific effects must be addressed properly in order to remove any technical artifacts and to be able to formulate biologically sound conclusions. The problem of probe effects becomes more pronounced as the density of tiling increases, as one does not have the option of selecting probe sequences for similar melting temperature, or when the tiled regions predominantly cover promoter regions, which are known to be GC-rich. Our method of standardization explicitly accounts for such sequence-specific biases and inter-array variability. Together with the accompanying robust peak-detection algorithm, MA2C's standardization procedure is especially important for data sets with a significant noise level - for instance, stemming from PCR amplification, which tends to increase probe effects.

### Normalization revisited

One issue we have not discussed so far is adjusting for the copy-number of probes or cross-hybridization of DNA with similar sequences. We chose not to model the sequence copy-number because both NimbleGen and Agilent use sufficiently long probes and also usually exclude repeat regions from their array design.

It is also instructive to note why our normalization method in equation 1 or equation 3 (See Materials and methods) gives a higher weight to the probes that are highly correlated between the two channels. Relying on the fact that the probes are long, NimbleGen tends to wash their arrays rather harshly after hybridization, minimizing cross-hybridization but also possibly leaving behind only random noise and causing a low correlation in low-GC probes between the two channels. Thus, as illustrated in Figures 2 and 3a, the low-GC probes are mostly measuring the background noise and also show a low inter-channel correlation; this relation between low intensity distribution and low inter-channel correlation in low-GC bins is the motivation behind MA2C's normalization method.

### Epilogue

**Figure 5:**
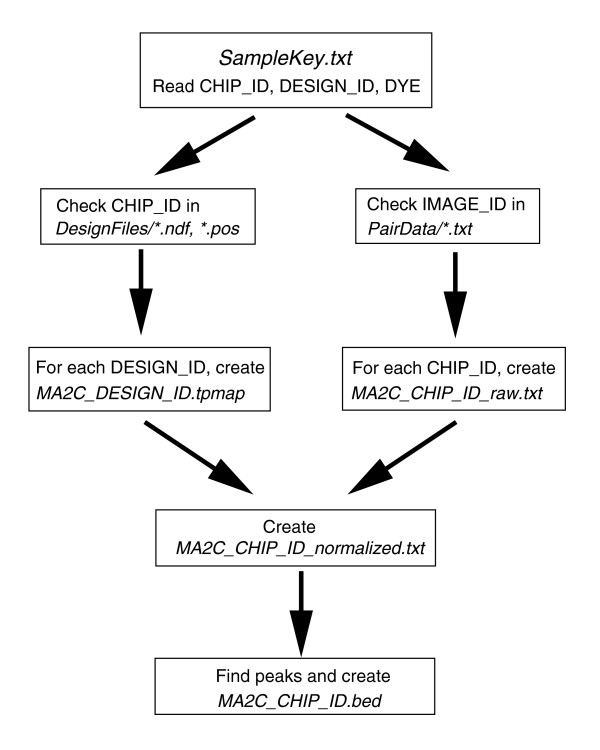
Workflow chart of MA2C. MA2C is fully automated and performs the tasks as shown.

MA2C is a novel model-based approach to analyzing two-color tiling microarray data, incorporating sequence-specific probe effects and powerful peak detection algorithms. The organization of MA2C's core functions is summarized in Figure 5. The GC-based normalization method can also be generalized to other long-oligonucleotide microarray applications, such as array-CGH and expression profiling. MA2C is also compatible with isothermal designs, where probe bias may be reduced but nevertheless still present. We have shown that the overall performance of MA2C is better than other currently available software. In addition to an easy, user-friendly interface, MA2C also provides informative graphical summaries of statistical analyses for array quality control. As ChIP-chip and other ways of studying chromatin structure become widespread common tools in biology, a program that can reliably analyze single or replicate experiment data from two-color microarrays will be a welcome contribution to the growing field.

## Materials and methods

### Normalization

We propose a normalization procedure that standardizes the data by modeling the GC-specific background hybridization intensities. Given an array, let *p*_*i *_denote its *i*^th^-probe and define *GC*_*i *_to be the total number of G and C nucleotides in *p*_*i*_. Denote the paired single channel log-intensities of *p*_*i *_as (*x*_*i*1_, *x*_*i*2_), where *x*_*i*1 _corresponds to the control and *x*_*i*2 _the treatment. Henceforth, let *i *index the probes, *j *the channels, and *k *the GC content bins. Then, our model assumes that the log-intensities (*x*_*i*1_, *x*_*i*2_), *i *∈ {*i*|*GC*_*i *_= *k*}, follow a bivariate distribution with GC-specific means (*μ*_1*k*_, *μ*_2*k*_), variances ($\sigma_{1k}^{2}$, $\sigma_{2k}^{2}$), and covariance *ξ*_*k *_between the two channels. Also implicit in the model is that although different GC bins are allowed to have different proportions of non-background probes, the signals of non-background probes are shifted across GC bins by the same mean, variance, and covariance as the background. Based on these assumptions, our model combines the single channel log-intensities to form a normalized, correlation weighted log-ratio *t*_*i *_as follows:

$$t_{i}=\frac{\left(x_{i2}-x_{i1})-({\hat{\mu }}_{2k}-{\hat{\mu }}_{1k}\right)}{\sqrt{{\hat{\sigma }}_{1k}^{2}+{\hat{\sigma }}_{2k}^{2}-2{\hat{\xi }}_{k}}}$$

where the parameters can be simply estimated as:

$${\hat{\mu }}_{jk}=\sum_{\left\{i|GC_{i}=k\right\}}\frac{x_{ij}}{n_{k}},$$

$${\hat{\sigma }}_{jk}^{2}=\sum_{\left\{i|GC_{i}=k\right\}}\frac{{\left(x_{ij}-{\hat{\mu }}_{jk}\right)}^{2}}{n_{k}},$$

and

$${\hat{\xi }}_{k}=\sum_{\left\{i|GC_{i}=k\right\}}\frac{\left(x_{i1}-{\hat{\mu }}_{1k})(x_{i2}-{\hat{\mu }}_{2k}\right)}{n_{k}},$$

where *n*_*k *_is the number of probes with *GC *= *k*. We further scale the *t*-values globally so that the rescaled *t*-values have variance 1.

**Figure 6:**
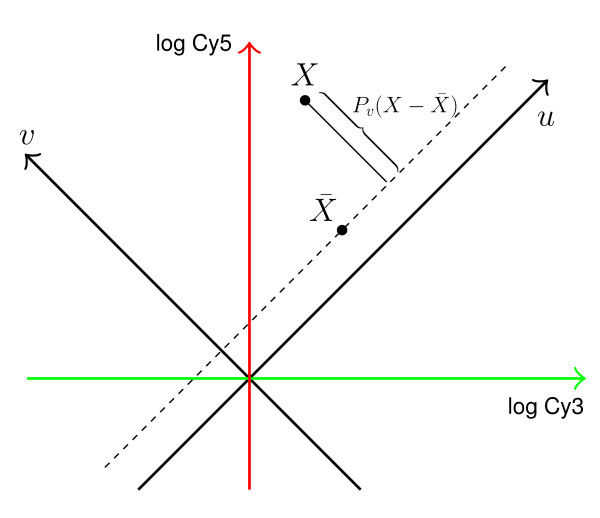
Geometrical interpretation of the normalization method. Our method first subtracts the baseline from log intensity vectors within each GC bin and then projects the adjusted vectors onto *v*-axis, yielding log mean-scaled ratios of the Cy5 and Cy3 signals within each GC bin. Finally, the projected values are adjusted for variance.

This method has the following geometrical interpretation as seen in Figure 6: assuming that Cy3 is the control and Cy5 the treatment channel, let {*e*_1_, *e*_2_} define an orthonormal basis of *R*^2^, where each probe *p*_*i*_, with log intensities *x*_*i*1 _= *log *(*Cy*3_*i*_) and *x*_*i*2 _= *log *(*Cy*5_*i*_), corresponds to a point *X*_*i *_= *x*_*x*1 _*e*_1 _+ *x*_*i*2 _*e*_2 _∈ *R*^2^. Define a new orthonormal basis {*u*, *v*}, where *u *= (*e*_1 _+ *e*_2_)/$\sqrt{2}$ and *v *= (*e*_2 _- *e*_1_)/$\sqrt{2}$ are obtained by rotating the original coordinate system by 45 degrees; and, define a projection operator *P*_*v*_: *R*^2 ^→ *R *onto *v*-axis as *P*_*v *_(*X*_*i*_) = (*x*_*i*2 _- *i*_*x*1_)*v*/$\sqrt{2}$. The projected vector thus measures the difference between log control and treatment signals. Let ${\bar{X}}_{i}$ be the average of all vectors in the GC bin to which *p*_*i *_belongs. We now consider *Z*_*i *_: = *P*_*v *_(*X*_*i *_- ${\bar{X}}_{i}$), which is just a dye-bias adjusted log-ratio, and finally define our normalized score as:

$$t_{i}:=Z_{i}/\sqrt{var(Z_{i})}.$$

The *t*-values thus yield log-ratios adjusted by the mean and normalized by the standard deviation within each GC bin.

Note that in equation 1, the covariance term *ξ*_*k *_has the effect of amplifying the difference between experiment and control probe intensities in GC bins that have a high baseline correlation between the two channels, while suppressing the difference in GC bins with low correlation. Therefore, the log-fold changes *x*_*i*2 _- *x*_*i*1 _are given more weight in GC bins with high correlation *ξ*_*k *_between the two channels than in low-correlation GC bins.

**Figure 7:**
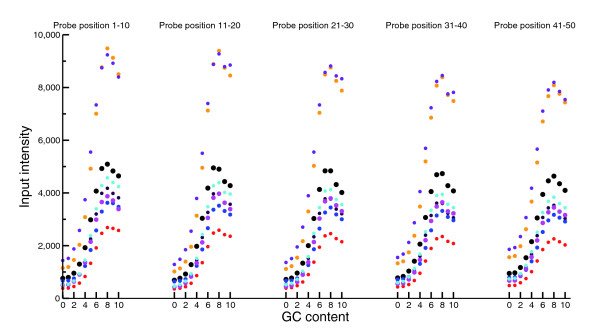
Average intensities of the control channel data from [12] as a function of position-specific GC counts. Each 50-mer probe is partitioned into 5 equal parts of 10 nucleotides, and average intensities are computed as a function of GC counts in each part. Different colors represent different samples. The GC-related variations of intensities behave similarly across the five locations on probes, and we thus see that the GC effect is not position specific.

We have checked that more complicated normalization methods based on position-specific ACGT effects, as in [1], dinucleotides or individual G and C counts yield results that are quite similar to the above simple and effective method (Figure 7).

### Robust estimation of parameters

With data symmetric in the two channels, the estimators given in equation 2 for *μ*_*jk*_, $\sigma_{jk}^{2}$, and *ξ*_*k *_should work very well. However, microarray data often tend to be skewed in one channel, even on the log scale, and the simple estimators can be sensitive to outliers. For this reason, we have developed a robust method for estimating these parameters. Our method generalizes Tukey's theory of bi-weight estimation, which is very robust for skewed data and has been successfully applied to microarray data previously [22]. In one dimension, Tukey's bi-weight estimation proceeds as follows: define a scaled distance *d*_*i *_between each data point *x*_*i *_and the current mean estimate μ* as:

$$d_{i}=\frac{x_{i}-\mu^{*}}{C\times M},$$

where *C *is a fixed constant and *M *= median_*i *_|*x*_*i *_- μ*|, the median absolute distance. We then calculate the bi-weight for each data point as *w*_i _= (1 - $d_{i}^{2}$)^2 ^for -1 ≤ *d*_*i *_≤ 1 and *w*_*i *_= 0 otherwise. Then, the mean is re-estimated as $\mu^{*}=\sum_{i}w_{i}x_{i}/\sum_{i}w_{i}$, and the process is repeated until a certain convergence criterion is satisfied.

We generalize the above approach to two dimensions and develop a similar procedure for estimating the parameters in equation 1 within each GC bin by using the elliptical or Mahalanobis distance given by:

$$d_{i}=\frac{Z_{i}^{t}\Sigma^{-1}Z_{i}}{(\sigma_{1k}^{*2}\sigma_{2k}^{*2}-\xi_{k}^{*2})\times C\times M}.$$

where:

$$Z_{i}^{t}\Sigma^{-1}Z_{i}:=\sigma_{2k}^{*2}{\left(x_{i1}-\mu_{1k}^{*}\right)}^{2}+\sigma_{1k}^{*2}{\left(x_{i2}-\mu_{2k}^{*}\right)}^{2}-2\xi_{k}^{*}(x_{i1}-\mu_{1k}^{*})(x_{i2}-\mu_{2k}^{*})$$

and $M=median_{i}|Z_{i}^{t}\Sigma^{-1}Z_{i}|$. Here, *Z*_*i *_is the projected vector previously described and Σ its variance matrix. In each iteration step, the mean is estimated as before, the variance as $\sigma^{*2}=\sum_{i}w_{i}{\left(x_{i}-\mu^{*}\right)}^{2}/\sum_{i}w_{i}$, and likewise for the covariance. Strictly speaking, the variance and covariance computed in this way are not consistent estimators, but as shown in the Results section, they do provide reasonable estimates of the parameters requisite for standardizing the data within and across arrays.

### Detection of peak regions

To detect peak regions, we have implemented several adaptations of the powerful window-based approach proposed by Johnson *et al*. [1] for Affymetrix tiling arrays. More precisely, we consider a sliding window of some user-defined length (400 bp to 1,000 bp) centered at each probe. A MA2Cscore is assigned using a user-selected scoring function based on median, pseudo-median, median polish, or trimmed mean of the probes in the window. The median and trimmed mean options are implemented by calculating the median or trimmed mean of all the probes in the window; when replicates are available, the median *t*-value or trimmed mean of all pooled probes in identical windows across replicates is used. The pseudo-median of a distribution is the median of all pairwise arithmetic means, as discussed in [23]. Median polish has been successfully applied in robust multi-chip analysis for Affymetrix gene expression arrays [24]. We recommend using median polish for experiments with a large number of replicate samples, while trimmed mean is recommended for arrays with densely tiled probes. The pseudo-median and median provide robust alternatives that can be applied in experiments that are not densely tiled and have few available replicates.

To compare the performance of different scoring functions, the triplicate H3 acetylation data at 38 bp spacing from [25] were analyzed using a window size of 1,000 bp and a *p *value cutoff of 10^-3^. Median polish gave the most number of peaks while trimmed mean gave the least, the difference in number being around 3%. The agreement among median, pseudo-median and trimmed mean was around 97-99%, while median polish agreed with other methods by 93-97%. Comparable results were obtained, with 1-2% less agreement, when the data were re-analyzed at 76 bp spacing by skipping probes. The best agreement was found between trimmed mean and pseudo-median at 99-100% while the worst agreement was between median and median polish at 90-93%.

To increase reliability, windows containing less than *k *probes are discarded, where *k *is again defined by the user. Just like MATscores, MA2Cscores approximately follow a normal distribution, with the representative scores of peak regions corresponding to the right tail. This fact easily allows us to assign a *p *value to each MA2Cscore using the normal probability distribution. The lower-bound of MA2Cscores for determining peaks may be based on either false discovery rate (FDR) or *p *value computations. As in [1], we empirically estimate FDR as follows: for a given cutoff value *M *> 0 of MA2Cscore, we find all peaks with MA2Cscore greater than *M *and all peaks with MA2Cscore less than -*M*. Then, FDR is estimated as #(negative MA2Cscore peaks)/#(positive MA2Cscore peaks), and the number of true positive peaks as #(positive MA2Cscore peaks) - #(negative MA2Cscore peaks). The FDR table, along with other informative histograms, is generated by MA2C.

### Implementation

We have implemented our method as a user-friendly, stand-alone Java package called MA2C, which is fully automated and only requires the user to select the directory path and treatment channels.

The file structure of NimbleGen data consists of three main components, *DesignFiles/, PairData/*, and *SampleKey.txt*, which should all reside in the same parent directory. The text file *SampleKey.txt *contains the relevant design information about individual arrays; in particular, the file must contain DESIGN_ID, CHIP_ID and DYE for each array. The directory *DesignFiles/*contains the sequence and position files corresponding to each DESIGN_ID, while *PairData/*contains the single channel data for each CHIP_ID. Even though MA2C is primarily designed for NimbleGen arrays, we have also successfully tested the program on Agilent data by reformatting the necessary files and obtained excellent results.

When the user begins by selecting *SampleKey.txt*, MA2C reads the file and displays the content in a table. If *DesignFiles/*and *PairData/*are present in the parent path, MA2C also automatically lists the directory contents in two separate tables; otherwise, the user has to choose the corresponding folder locations. The user then selects the treatment channel for each experiment to be analyzed and clicks the Run button, which prompts MA2C to perform the normalization and peak detection steps as follows.

#### Step 1: *DesignFiles*/

For each DESIGN_ID, MA2C automatically reads the corresponding *.ndf *and *.pos *files and generates a *.tpmap *file containing the sequence, chromosome, position, and array coordinate information of probes.

#### Step 2: *PairData*/

For each chosen treatment channel with given CHIP_ID and DYE, MA2C searches for the correct two-channel data files. It is thus important that the pair data files contain a column corresponding to IMAGE_ID. For fast future access and also for compressed storage, the program combines each two-channel data into a single file named *MA2C_CHIP_ID_raw.txt*. Normalized data are similarly stored in files with the extension *_normalized.txt*.

#### Step 3: *MA2C_output*/

MA2C automatically creates this directory for writing files used in quality control of normalization and peak detection steps. The enriched regions are output in both *.xls *and *.bed *files that contain the chromosome, start, end, *p *value, MA2Cscore and peak-center information for each detected peak. *MA2Cscore.bar *and *ratio.bar *files are created for visualization using Affymetrix's Integrated Genome Browser [26].

MA2C is an open source Java package that can be downloaded from [27]. MA2C runs on all platforms that support Java Runtime Environment 5.0 or higher and has been successfully tested on OS X, Linux and Windows operating systems. The program is written so as to economize the size of required files; once the *.tpmap *and *_raw.txt *files have been created, the subsequent runs of MA2C will use only those files and the user may remove the *.ndf*, *.pos*, and other pair data files. This approach can save hundreds of megabytes of disk space. In addition, our program is fast, the total execution time being usually less than a couple of minutes for multiple arrays. For example, on a laptop with a 2.13 GHz Intel M processor and 2 GB RAM, it takes 18 seconds to build a sequence file for 370,000 probes, 16 seconds to normalize the raw data, and 14 seconds to find peaks.

## Abbreviations

bp, base-pairs; ChIP, chromatin immunoprecipitation; FDR, false discovery rate.
